# Death of Dr. Samuel George Morton

**Published:** 1851-07

**Authors:** 


### DEATH OF DR. SAMUEL GEORGE MORTON.

We have just heard of the death of this eminent physician and naturalist. The melancholy event will be deplored by all who take an interest in the progress of science in America. Dr. Morton had for many years devoted himself with great assiduity to the cultivation of natural history without relaxing in his devotion to medicine. He has left behind him the largest collection of human skulls in our country, if not in the world, and in his great work, the *Crania Americana*, leaves a monument of industry and research which has placed his name among the first of ethnologists. Y.

